# Molecular Epidemiology of *Anisakis* spp. in Wedge Sole, *Dicologlossa cuneata* (Moreau, 1881), from Fishmarkets in Granada (Southern Spain), Caught in Two Adjacent NE and CE Atlantic Areas

**DOI:** 10.3390/pathogens10101302

**Published:** 2021-10-11

**Authors:** Sara Buzo-Domínguez, Manuel Morales-Yuste, Ana María Domingo-Hernández, Rocío Benítez, Francisco Javier Adroher

**Affiliations:** Departamento de Parasitología, Facultad de Farmacia, Universidad de Granada, 18071 Granada, Spain; sarabuzod@gmail.com (S.B.-D.); yuste@ugr.es (M.M.-Y.); anamariadomingohernandez@gmail.com (A.M.D.-H.); rbenitez@ugr.es (R.B.)

**Keywords:** wedge sole, *Dicologlossa cuneata*, anisakiasis, *Anisakis*, *Hysterothylacium*, Andalusia, Spain, FAO 27.IXa, Morocco, FAO 34.1.11

## Abstract

The presence of third stage larvae (L3) of *Anisakis* spp. in wedge sole, *Dicologlossa cuneata* (Moreau, 1881), purchased in fishmarkets in the city of Granada (Andalusia, southern Spain) was assessed. The wedge sole were caught in two FAO zones: area 27.IXa NE Atlantic (SW Spain coast) and area 34.1.11 CE Atlantic (NW Morocco coast). Only *Anisakis* larvae, type I, were detected in the largest fish (>20 cm) from the CE Atlantic. These were molecularly identified as *A. simplex* s.s. The prevalence (P) of *Anisakis* in this area was 12.5% and the mean intensity (MI) was 1. The presence of *Hysterothylacium* spp. larvae was also detected in the fish from both areas, with the prevalence being approximately double in the CE Atlantic area (12.5 vs. 5.7). A comparison between the *Anisakis*-infected and non-infected fish from this area showed that the former were significantly longer than the latter (*p* < 0.01). These results show that *Anisakis* parasitization of wedge sole sold in the markets of the city of Granada is of low prevalence and intensity (P = 4.5, MI = 1), especially in those from area 27.IXa (P = 0), indicating that the risk of human infection is low, particularly as this fish is traditionally prepared by deep-frying in oil in Andalusia (southern Spain).

## 1. Introduction

Anisakids are parasitic nematodes whose life cycle includes an enormous variety of fish, mainly marine, which can act as hosts for their third stage larvae (L3). Some of these nematodes may be ingested by humans on consuming fish and/or squid containing viable larvae and may result in anisakiasis/anisakidosis. The causative agents most frequently identified are those of the genera *Anisakis* (~97%) and *Pseudoterranova* (~3%) [1,2,3]. The most frequent etiological agent is *Anisakis simplex* s.l. The ingestion of the viable larvae of these parasites can lead to the appearance of digestive symptoms (intense pain which may be accompanied by nausea, vomiting, diarrhoea, etc.), allergic symptoms (urticaria, angioedema, etc.) or both in what is known as gastroallergic anisakiasis [1,2,3]. Many studies have identified these parasites in fish belonging to a wide range of orders and families. However, few of these studies have involved the Pleuronectiformes (flatfish) [4,5,6,7,8,9,10], particularly those of the family Soleidae [11,12,13,14,15].

The wedge sole, *Dicologlossa cuneata* (Moreau, 1881), despite being a commercially important species in some areas and especially in Spain and Portugal, has been rarely studied. It is a demersal marine fish which can also live in brackish water and is usually found at depths of between 10 and 150 m, although it has been recorded at 460 m. Belonging to the family Soleidae (sole) it is found all along the Eastern Atlantic coast from the Bay of Biscay to South Africa and is also present in some parts of the Mediterranean.

It is highly prized in Andalusia (southern Spain), particularly around the Gulf of Cádiz, where more than 225,000 kg of wedge sole were landed in 2020 [16]. The aim of the present study was to identify the anisakid species they contain in order to determine whether the wedge sole on sale in southern Spain represent a risk to consumer health. The fishmarkets of the city of Granada were chosen for this purpose. The concurrence of fish from two different but adjacent FAO catch areas has provided an opportunity to compare them in terms of anisakiasis risk to the consumer.

## 2. Results

### 2.1. Host

The 110 wedge sole examined ranged from 15.0–26.6 cm in length and from 21.8–170.0 g in weight while the condition factor (CF) was from 0.56–1.11 (Table 1). The relationship between weight (W) and total length (L) resulted in a potential line with an exponent close to 3 (W = 0.0014 · L^3.5546^; coefficient ± 0.0003 (SD) and exponent ± 0.0754 (SD), R² = 0.9439]) implying a cubic relationship as generally considered in the literature for fish [17,18]. Statistical comparison of the length, weight and CF of the fish revealed significant differences for these parameters between the two areas sampled (Table 1, *p* << 0.001), with those from area 27.IXa having the lower values.

### 2.2. Morphological and Molecular Identification. Epidemiological Parameters

Morphological identification revealed five L3 *Anisakis* type I (sensu Berland [19]) and six L3 and four L4 of *Hysterothylacium* spp. in the fish. The five larvae of *Anisakis* were found on the surface of the fish, sometimes attached to them as if abandoning the host while of the 10 larvae of *Hysterothylacium* spp., six were found in the visceral cavity and four following pepsic digestion of the viscera. No fish was parasitized by both ascaridoids at the same time and only one fish contained more than one larva (a wedge sole from the Atlantic coast of Morocco with two L3 of *Hysterothylacium*). Table 1 shows the total epidemiological parameters, revealing the prevalence of ascaridoids to be 12.7%: 4.5% for *Anisakis* spp. and 8.2% for *Hysterothylacium* spp. Of the 110 wedge sole studied, 70 were from the Spanish coast (FAO 27.IXa, NE Atlantic) and 40 from the northwestern coast of Morocco (FAO 34.1.11, CE Atlantic). Analysis of the epidemiological parameters by area revealed that although there was a lower occurrence of *Hysterothylacium* spp. in area 27.IXa, the difference between the areas was not significant (*p* = 0.28). On the other hand, *Anisakis* spp. was only detected in area 34.1.11 (*p* < 0.01), where the larger fish were found (*p* < 0.004) (Table 1 and Table 2). All the larvae of *Anisakis* were subjected to a molecular genetic study by PCR-RFLP and identified as A. *simplex* s.s.

Finally, the length, weight and CF of parasitized and non-parasitized fish in the two areas studied were compared (Table 3). In area 27.IXa only significant differences in length were observed, with the fish parasitized by *Hysterothylacium* being larger (*p* < 0.01). However, these differences were not found in area 34.1.11. Analysis of the parasitization by *Anisakis* in this area of the Central Atlantic, the only area where this parasite was found, showed significant differences in weight (*p* = 0.05) and length (*p* < 0.01) but not in CF (Table 3).

## 3. Discussion

The wedge sole is a fish of high commercial value in the coastal areas where it is found and is traditionally exploited by a fleet of bottom-trawl and gillnet boats. It tends to be found on sand or mud substrates where it feeds on a wide variety of small benthic organisms, principally crustaceans, polychaetes and bivalve molluscs [20]. Belghyti et al. [21] reported that in *D. cuneata* benthic or epifaunal animals, especially amphipods of which up to 16 species were found in stomach contents, polychaete annelids constitute the main part of the diet, regardless of the age of the fish. The smaller prey animals, such as brachyurae, mysids and cumaceans tend to disappear from the diet of older fish [22]. These feeding habits may serve to explain ascaridoid infestation, particularly that by *Hysterothylacium* spp., since they use invertebrates as their first intermediate host, particularly crustaceans (copepods and amphipods, and less often, mysids, isopods, decapods and euphausiids) with some chaetognaths, ctenophores, cnidarians and echinoderms (see [23] for references). *Anisakis* spp. generally employs euphausiids and large calanoid copepods as their first intermediate host (see [3] for references), although other invertebrates have occasionally been described in the literature. The largest planktonic or benthic invertebrates (crustaceans, chaetognaths and polychaetes) and fish can act as transport hosts for both parasites. However, the most frequent hosts do not appear to form a major part of the wedge sole’s diet. This may explain the low prevalence and intensity of the two parasites in our fish. Nonetheless, the present study shows that in *Anisakis*, but not in *Hysterothylacium*, there is a relationship between catch area and parasitization (Table 1). This is particularly relevant as an increasing number of studies relate the risk of infection of fish by *Anisakis* with the catch area [24,25,26,27,28]. It is also true that in many cases a direct relationship has been reported between fish size and prevalence [25,28,29], suggesting a greater probability of ingestion of *Anisakis* with increasing fish age, although this does not occur in all species of fish [30,31,32] possibly due to changes in diet, to immune system activity or to a greater mortality of fish with a high intensity of parasitization [31,33,34]. In the present study a greater prevalence in the larger fish was also observed (Table 2), corresponding to the catch area 34.1.11. It is not possible to state whether this correlates more strongly with the catch area or fish size, although it seems likely that both factors were involved simultaneously. Various possible causes have been suggested to relate infection rates with the catch area, such as a greater presence of both intermediate and definitive hosts which would favour the life cycle of the parasites. Another studies, relating infection levels to fish size, consider the accumulation of parasites throughout the life of the host as it has been established that *Anisakis simplex* s.l. can survive for between 3–5 years in the host fish [34,35]. In the case of the wedge sole, its larger size facilitates the capture of larger prey (such as euphausiids, chaetognaths, polychaetes or small fish) and/or greater quantities of prey with the concomitant increase in the probability of infection, as observed in the present study (Table 2 and Table 3). 

Fulton’s Condition Factor (CF) for fish [17] has been considered to be an indicator of general health [36]. However, infection by *Anisakis* does not appear to have affected it in the wedge sole sampled (Table 1 and Table 3). Although there is some controversy regarding the effects of parasitization on CF, the intensity in the present study was so low as to be unlikely to affect this parameter and the differences found are more likely due to the degree of development of the fish, with the smallest having the lowest CF (Table 1). It is possible that the CF is affected when the parasitization intensity is high (or as suggested by Serrat et al. [37], it will be affected when the parasite burden affects the availability of energy for the fish) and by other factors such as season, age/length or maturity of the fish ([34,38], see also [39] and references), as may be the case in the present study. However, other authors have not found any relationship between parasitization and CF [25,29,34,40].

**Figure 1:**
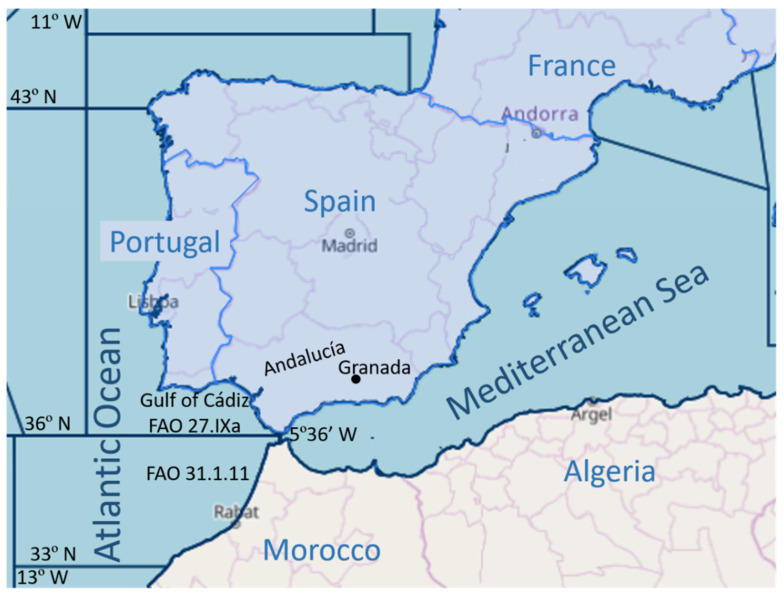
Map indicating the FAO fish catch areas of the sampled wedge sole in the Atlantic Ocean and the fishmarkets of Granada (●) in which the presence of *Anisakis* in this fish host has been surveyed.

Few studies have been carried out to investigate the parasitization of wedge sole by anisakids and these appear to have been limited to catch area 27.IXa, where *Anisakis*, but not *Hysterothylacium*, were recorded. In Spain, De la Torre Molina et al. [41] found none in a sample of 35 wedge sole, probably caught in the Gulf of Cádiz, from a fishmarket in the province of Córdoba (Andalusia). Neither did Silva and Eiras [15] find any anisakids in a sample of 25 wedge sole from the west coast of Portugal. At a later date, Marques et al. determined the prevalence of *Anisakis* spp. in wedge sole all along the Portuguese coast, finding larvae of the genus *Anisakis* in the north (P = 2.5%; 160 fish) but none in the centre (175 fish) or south (155 fish) [12], as occurred in the present study for the coast of the Gulf of Cádiz, adjacent to the south coast of Portugal (Figure 1). These authors identified the larvae as *A. pegreffii* [13]. However, the fish from the northern coast of Morocco (catch area 34.1.11) showed a prevalence of 12.5% for larvae of *Anisakis* type I (sensu Berland [19]), although always at a minimal intensity (Table 1). It has not been possible to locate data for this catch area in the literature to compare with the results of the present survey, although studies on other fish species have confirmed the presence of *Anisakis* spp. in the Atlantic waters of Morocco [42,43,44]. With regard to the larvae of *Anisakis* attached externally to the wedge sole, this may be due to a possible migration of the larvae towards the exterior following the death of the host, as reported for anchovies [24,45]. It should also be remembered that these wedge sole were from Morocco and had thus been dead for longer, as mentioned previously.

Genetic analysis of the larvae of *Anisakis* collected from the wedge sole from catch area 34.1.11 revealed all to be *A. simplex* s.s. Although previous studies of other fish species in this area reported finding both *A. simplex* s.s. and *A. pegreffii* as well as hybrids between the two [42,46], the hypothesis that the former is principally benthic and the latter pelagic [47,48] appears to be supported by the presence of only the former in the wedge sole, which are a benthic fish. Furthermore, Mattiucci et al. [49] established the southern limits of the territory of *A. simplex* s.s. at 35° N, roughly the catch area (northern Atlantic coast of Morocco, between 33° N and 36° N) in zone FAO 34.1.11 (Figure 1).

This survey shows that the wedge sole sold in the fishmarkets of Granada have a low prevalence and medium intensity of the larvae of *Anisakis* (P = 4.5%, MI = 1), implying a low risk of infection to the consumer. Furthermore, the most highly prized and widely available wedge sole in the city is that from the Gulf of Cádiz, where no *Anisakis* spp. were detected, thus representing no risk of anisakiasis to the consumer. In addition, wedge sole from the northern Atlantic coast of Morocco, despite containing *Anisakis* (P = 12.5%), present only a low risk of infection due to a minimal intensity and the culinary tradition in Andalusia of deep-frying the fish in oil.

## 4. Materials and Methods

### 4.1. Hosts and Search for Parasites

We randomly selected 110 wedge sole from two large fishmarkets in the city of Granada (Andalusia, southern Spain) between December 2020 and March 2021, recording their origin. The majority (65.4%) were caught in the Gulf of Cádiz, FAO area 27.IXa (SW Spain coast, Figure 1), and landed in the ports of the area (Isla Cristina, Sanlúcar de Barrameda and Bahía de Cádiz). Some of the fish purchased (34.6%) had been imported from Morocco (caught in FAO zone 34.1.11, NW Morocco coast, Figure 1), which involved an interval of more than 24 h between catching and sampling, unlike the fish from the Gulf of Cádiz (less than 24 h between catching and sampling). The fish were kept refrigerated while being transported to the laboratory, where, after identification as *Dicologlossa cuneata* (Moreau, 1881), their weight and total length was recorded. The Fulton’s condition factor (CF) of the fish, an index of the apparent health of the fish [36], was calculated using the formula CF = 100 × W/L^3^, where W = total weight (g) and L = total length (cm) [17,18]. Each specimen was examined externally for possible macroparasites and, after washing with tap water, was dissected for parasitological study, separating the musculature and viscera and examining them thoroughly for nematodes. After this examination, the viscera and musculature of each fish were subjected separately to pepsic digestion at pH 2.0, at 37 °C as described previously [25] for 1 h for the former and 3 h for the latter. Next, the remains of the digestion were carefully examined to detect any possible nematodes. The ascaridoids found were then morphologically identified to the genus level [19,50,51,52].

### 4.2. Genetic Identification of Anisakis spp. Larvae

For the genetic identification of the larvae, polymerase chain reaction followed by restriction fragment length polymorphism (PCR-RFLP) of the ribosomal fragment ITS1-5.8S-ITS2 was carried out. Each larva was individually prepared for DNA isolation using a commercial RealPure kit according to the manufacturer’s instructions. The selected rDNA fragment was amplified using the primers: NC5 (forward) 5′ GTAGGTGAACCTGCGGAA GGATCATT 3′, and NC2 (reverse) 5′ TTAGTTTCTTTTCCTCCGCT 3′, described by Zhu et al. [53]. Amplification was carried out with the following programming: one cycle of 94 °C for 5 min, 60 °C for 30 s, 72 °C for 90 s; 35 cycles of 94 °C for 30 s, 60 °C for 30 s, 72 °C for 60 s; and a final cycle at 94 °C for 30 s, 60 °C for 30 s and 72 °C for 5 min, then cooled and kept at 4 °C until use. The expected size of the amplified fragment was around 1000 bp. RFLP of the amplicons was then performed with the restriction enzymes *TaqI* and *HinfI* (Fast Digest), used individually at a final concentration of 0.5 U/μL and at temperatures of 65 °C and 37 °C, respectively, for 10 min. A 3% agarose gel electrophoresis was performed to visualise the banding patterns of the *Anisakis* type I larvae studied, in order to determine the species [54,55]. *TaqI* digestion controls of the *A. simplex* s.s. DNA amplicon produced 3 fragments of 430, 400 and 100 bp, while those of the *A. pegreffii* produced 3 bands of 400, 320 and 150 bp. When digested with the *HinfI*, the fragments were 620, 250 and 100 bp for the *A. simplex* s.s. and 370, 300 and 250 bp for the *A. pegreffii*.

### 4.3. Epidemiological Parameters and Statistical Comparisons

Epidemiological parameters of prevalence (P), mean intensity (MI) and mean abundance (MA), as defined by Bush et al. [56], were calculated and compared using the free Quantitative Parasitology 3.0 software [57] to cope with notoriously left-skewed parasite frequency distributions, based on the theoretical work of Rózsa et al. [58] and Reiczigel et al. [59]. Differences in prevalence were assessed using Fisher’s exact test. A bootstrap 2-sample *t*-test (with 20,000 repetitions) was used to compare mean intensities and mean abundances. ANOVA and, when significant, the Student’s *t*-test were used for the statistical comparison of parasitization of fish of different length classes. As wedge sole maturity occurs in the length range of 17–19.9 cm [60], the fish were grouped into three length groups for study: <17 cm or immature fish; 17–19.9 cm or fish in the period of completing maturity; and ≥20.0 cm or fully mature fish. The Student’s t-test was used for a pairwise comparison of length, weight and condition factor of the fish.

## Figures and Tables

**Table 1:** Epidemiological parameters of *Anisakis* spp. and *Hysterothylacium* spp. infection, total and by catch zones, in wedge sole sampled in fishmarkets in Granada (Spain).

	Parameters	Both Zones	Zone 27.IXa	Zone 34.1.11
Host				
Wedge sole	No. of fish (F)	110	70	40
	Mean weight ± SD (range)	57.9 ± 38.0 (21.8–170.0)	34.2 ± 6.8 (21.8–56.5)	99.4 ± 34.4 *** (48.4–170.0)
	Mean length ± SD (range)	19.2 ± 3.1 (15.0–26.6)	17.7 ± 1.1 (15.0–19.6)	22.8 ± 2.0 *** (18.5–26.6)
	Condition factor ± SD (range)	0.72 ± 0.11 (0.56–1.11)	0.67 ± 0.07 (0.56–0.94)	0.81 ± 0.11 *** (0.60–1.11)
**Parasites**				
Ascaridoids	Prevalence (%) CI 95%	12.7 0.08–0.20	5.7 1.6–14.0	25.0 ** 12.7–41.2
	Mean intensity (range) CI 95%	1.07 (1–2) 1.00–1.21	1 (1) uncertain	1.10 (1–2) ^ns^ 1.00–1.30
	Mean abundance CI 95%	0.14 0.07–0.21	0.06 0.01–0.11	0.28 * 0.13–0.43
*Anisakis* spp.	Prevalence (%) CI 95%	4.5 0.02–0.10	0 0.0–5.1	12.5 ** 4.2–26.8
	Mean intensity (range) CI 95%	1 uncertain	0 nd	1 (1) ^ns^ uncertain
	Mean abundance CI 95%	0.05 0.01–0.08	0 uncertain	0.13 * 0.03–0.22
*Hysterothylacium* spp.	Prevalence (%) CI 95%	8.2 0.04–0.15	5.7 1.6–14.0	12.5 ^ns^ 4.2–26.8
	Mean intensity (range) CI 95%	1.11 (1–2) 1.00–1.33	1 (1) uncertain	1.2 (1–2) ^ns^ 1.00–1.40
	Mean abundance CI 95%	0.09 0.04–0.15	0.06 0.01–0.11	0.15 ^ns^ 0.05–0.30

Weight in g, length in cm. Prevalence = 100·N/F, mean intensity = A/N, mean abundance = A/F; where F is the total number of fish, N is the number of infected fish, and A is the number of larvae. SD: standard deviation. CI: confidence interval. nd: not determined. A Student’s *t*-test comparison of the length, weight and condition factor between zones FAO 27.IXa and 34.1.11 showed high significance (*** *p* << 0.001). Comparison of the epidemiological parameters between zones: ** *p* < 0.01; * *p* < 0.05; ^ns^, not significant.

**Table 2:** Epidemiological parameters of *Anisakis* spp. and *Hysterothylacium* spp. infection, according to length classes, in wedge sole sampled in fishmarkets in Granada (Spain).

	Length (cm)	<17.0	17.0–19.9	≥20.0
Parasites	Fish number (F)	29	45	36
Ascaridoids	Prevalence (%) ^ns^ CI 95%	3.4 0.1–17.8	8.9 2.5–21.2	25.0 12.1–42.2
	MI (range) CI 95%	1 uncertain	1 uncertain	1.11 (1–2) 1.00–1.33
	MA CI 95%	0.03 0.00–0.10	0.09 0.02–0.18	0.28 0.11–0.44
*Anisakis* spp.	Prevalence (%) * CI 95%	0 0.0–11.9	0 0.0–7.9	13.9 4.7–29.5
	MI CI 95%	0	0	1 uncertain
	MA CI 95%	0	0	0.14 0.03–0.25
*Hysterothylacium* spp.	Prevalence (%) ^ns^ CI 95%	3.4 0.1–17.8	8.9 2.5–21.2	11.1 3.1–26.1
	MI (range) CI 95%	1 uncertain	1 uncertain	1.25 (1–2) 1.00–1.50
	MA CI 95%	0.03 0.00–0.10	0.09 0.02–0.18	0.14 0.03–0.31

Prevalence = 100·N/F; MI, mean intensity = A/N; MA, mean abundance = A/F; where F is the total number of fish, N is the number of infected fish, and A is the number of larvae. CI: confidence interval. ANOVA comparison of prevalence by length classes showed significance (* *p* < 0.004) for *Anisakis*; ^ns^, not significant.

**Table 3:** Length, weight and condition factor of uninfected and infected wedge sole sampled in the fishmarkets of Granada (Spain), by catch area.

FAO Area	Parameters		Ascaridoids ^#^	*Hysterothylacium* spp.	*Anisakis* spp.
27.IXa, NE Atlantic, coast of SW Spain	Fish weight ± SD	U I	33.7 ± 6.2 42.2 ± 12.0 ^ns^	33.7 ± 6.2 42.2 ± 12.0 ^ns^	ND
	Fish length ± SD	U I	17.1 ± 1.0 18.5 ± 1.4 **	17.1 ± 1.0 18.5 ± 1.4 **	ND
	Condition factor ± SD	U I	0.67 ± 0.07 0.65 ± 0.06 ^ns^	0.67 ± 0.07 0.65 ± 0.06 ^ns^	ND
34.1.11, CE Atlantic, coast of NW Morocco	Fish weight ± SD	U I	96.2 ± 33.5 109.1 ± 37.2 ^ns^	100.7 ± 34.6 90.8 ± 35.6 ^ns^	95.4 ± 33.3 127.3 ± 32.0 *
	Fish length ± SD	U I	22.5 ± 1.9 23.5 ± 2.3 ^ns^	22.9 ± 1.9 22.3 ± 2.6 ^ns^	22.5 ± 1.9 24.8 ± 1.2 **
	Condition factor ± SD	U I	0.81 ± 0.13 0.81 ± 0.12 ^ns^	0.81 ± 0.13 0.78 ± 0.11 ^ns^	0.81 ± 0.13 0.83 ± 0.13 ^ns^

^#^ The only ascaridoids detected in the fish from the Gulf of Cadiz (FAO 27.IXa) were of the genus *Hysterothylacium*. SD: standard deviation. U: uninfected fish. I: infected fish. ND: *Anisakis* not detected. Student’s t-test comparison of length, weight and condition factor between uninfected and infected fish: ** *p* < 0.01; * *p* = 0.05; ^ns^, not significant.

## Data Availability

The datasets generated during and/or analysed during the current study are all included in this manuscript.

## References

[1] Rello Yubero F.J., Adroher Auroux F.J., Valero-López A. (2004). Anisákidos parásitos de peces comerciales. Riesgos asociados a la salud pública. An. Real Acad. Cienc. Vet. Andal. Orient..

[2] Ishikura H., Kikuchi K., Nagasawa K., Ooiwa T., Takamiya H., Sato N., Sugane K., Sun T. (1993). Anisakidae and anisakidosis. Progress in Clinical Parasitology.

[3] Adroher-Auroux F.J., Benítez-Rodríguez R. (2020). Anisakiasis and *Anisakis*: An underdiagnosed emerging disease and its main etiological agents. Res. Vet. Sci..

[4] McClelland G., Misra R.K., Martell D.J. (1985). Variations in abundance of larval anisakines, sealworm (*Pseudoterranova*
*decipiens*) and related species, in Eastern Canadian cod and flatfish. Can. Tech. Rep. Fish. Aquat. Sci..

[5] Marques J.F., Santos M.J., Cabral H.N. (2009). Zoogeographical patterns of flatfish (Pleuronectiformes) parasites in the Northeast Atlantic and the importance of the Portuguese coast as a transitional area. Sci. Mar..

[6] McClelland G., Misra R.K., Marcogliese D.J. (1983). Variations in abundance of larval anisakines, sealworm (*Phocanema*
*decipiens*) and related species in cod and flatfish from the southern Gulf of St. Lawrence (4T) and the Breton Shelf (4Vn). Can. Tech. Rep. Fish. Aquat. Sci..

[7] Marques J.F., Santos M.J., Teixeira C.M., Batista M.I., Cabral H.N. (2011). Host-parasite relationships in flatfish (Pleuronectiformes)—The relative importance of host biology, ecology and phylogeny. Parasitology.

[8] Oliva M.E., Castro R.E., Burgos R. (1996). Parasites of the flatfish *Paralichthys*
*adspersus* (Steindachner, 1867) (Pleuronectiformes) from northern Chile. Mem. Inst. Oswaldo Cruz.

[9] González M.T., Acuña E., Oliva M.E. (2001). Metazoan parasite fauna of the bigeye flounder, *Hippoglossina*
*macrops*, from northern Chile. Influence of host age and sex. Mem. Inst. Oswaldo Cruz.

[10] Alarcos A.J., Pereira A.N., Taborda N.L., Luque J.L., Timi J.T. (2016). Parasitological evidence of stocks of *Paralichthys*
*isosceles* (Pleuronectiformes: Paralichthyidae) at small and large geographical scales in South American Atlantic coasts. Fish. Res..

[11] Álvarez F., Iglesias R., Paramá A.I., Leiro J., Sanmartín M. (2002). Abdominal macroparasites of commercially important flatfishes (Teleostei: Scophthalmidae, Pleuronectidae, Soleidae) in northwest Spain (ICES IXa). Aquaculture.

[12] Marques J.F., Santos M.J., Cabral H.N. (2006). Soleidae macroparasites along the Portuguese coast: Latitudinal variation and host–parasite associations. Mar. Biol..

[13] Marques J.F., Cabral H.N., Busi M., D’Amelio S. (2006). Molecular identification of *Anisakis* species from Pleuronectiformes off the Portuguese coast. J. Helminthol..

[14] Seesao Y. (2015). Caractérisation des Anisakidae Dans les Poissons Marins: Développement D’une Méthode D’identification Par Séquençage à Haut-Débit et Étude de Prévalence. Ph.D. Thesis.

[15] Silva M.E.R., Eiras J.C. (2003). Occurrence of *Anisakis* sp. in fishes off the Portuguese West coast and evaluation of its zoonotic potential. Bull. Eur. Assoc. Fish Pathol..

[16] IDAPES Consulta estadísticas pesqueras Primera Venta de Pesca Fresca en Lonja. http://www.juntadeandalucia.es/agriculturaypesca/idapes/.

[17] Fulton T.W. The rate of growth of fishes. Proceedings of the 22nd Annual Report of the Fishery Board of Scotland.

[18] Nash R.D.M., Valencia A.H., Geffen A.J. (2006). The origin of Fulton’s condition factor—Setting the record straight. Fisheries.

[19] Berland B. (1961). Nematodes from some Norwegian marine fishes. Sarsia.

[20] Quéro J.-C., Desoutter M., Lagardère F., Whitehead P.J.P., Bauchot M.-L., Hureau J.-C., Nielsen J., Tortonese E. (1986). Soleidae. Fishes of the North-Eastern Atlantic and the Mediterranean.

[21] Belghyti D., Berrada-rkhami O., Boy V., Aguesse P., Gabrion C. (1994). Population biology of two helminth parasites of flatfishes from the Atlantic coast of Morocco. J. Fish Biol..

[22] Belghyti D., Aguesse P., Gabrion C. (1993). Éthologie alimentaire de *Citharus*
*linguatula* et *Dicologoglossa cuneata* sur les côtes atlantiques du Maroc. Vie Milieu/Life Environ..

[23] Adroher-Auroux F.J., Benítez-Rodríguez R., Sitjà-Bobadilla A., Bron J.E., Wiegertjes G., Piazzon M.C. (2021). *Hysterothylacium* *aduncum*. Fish Parasites: A Handbook of Protocols for Their Isolation, Culture and Transmission.

[24] Rello F.J., Adroher F.J., Benítez R., Valero A. (2009). The fishing area as a possible indicator of the infection by anisakids in anchovies (*Engraulis*
*encrasicolus*) from southwestern Europe. Int. J. Food Microbiol..

[25] Molina-Fernández D., Malagón D., Gómez-Mateos M., Benítez R., Martín-Sánchez J., Adroher F.J. (2015). Fishing area and fish size as risk factors of *Anisakis* infection in sardines (*Sardina*
*pilchardus*) from Iberian waters, southwestern Europe. Int. J. Food Microbiol..

[26] Adroher F.J., Valero A., Ruiz-Valero J., Iglesias L. (1996). Larval anisakids (Nematoda: Ascaridoidea) in horse mackerel (*Trachurus*
*trachurus*) from the fish market in Granada (Spain). Parasitol. Res..

[27] Levsen A., Karl H. (2014). *Anisakis**simplex* (s.l.) in grey gurnard (*Eutrigla*
*gurnardus*) from the North Sea: Food safety considerations in relation to fishing ground and distribution in the flesh. Food Control.

[28] Brattey J., Bishop C.A. (1992). Larval *Anisakis*
*simplex* (Nematoda: Ascaridoidea) infection in the musculature of Atlantic cod, *Gadus*
*morhua*, from Newfoundland and Labrador. Can. J. Fish. Aquat. Sci..

[29] Molina-Fernández D., Rubio-Calvo D., Adroher F.J., Benítez R. (2018). Molecular epidemiology of *Anisakis* spp. in blue whiting *Micromesistius*
*poutassou* in eastern waters of Spain, western Mediterranean Sea. Int. J. Food Microbiol..

[30] Rello F.J., Valero A., Adroher F.J. (2008). Anisakid parasites of the pouting (*Trisopterus*
*luscus*) from the Cantabrian Sea coast, Bay of Biscay, Spain. J. Helminthol..

[31] Bussmann B., Ehrich S. (1979). Investigations on infestation of blue whiting (*Micromesistius*
*poutassou*) with larval *Anisakis* sp. (Nematoda: Ascaridida). Arch. Fischereiwiss..

[32] Davey J.T. (1972). The incidence of *Anisakis* sp. larvae (Nematoda: Ascaridata) in the commercially exploited stocks of herring (*Clupea*
*harengus* L., 1758,) (Pisces: Clupeidae) in British and adjacent waters. J. Fish Biol..

[33] Valero A., Martín-Sánchez J., Reyes-Muelas E., Adroher F.J. (2000). Larval anisakids parasitizing the blue whiting, *Micromesistius*
*poutassou*, from Motril Bay in the Mediterranean region of southern Spain. J. Helminthol..

[34] Eltink A. (1988). *Anisakis* larvae (Nematoda: Ascaridida) in mackerel, (*Scomber*
*scombrus* L.) in lCES sub-areas IV, VI, VII and VIII in 1970-1971 and 1982–1984. Int. Counc. Explor. Sea.

[35] Smith J.W. (1984). *Anisakis**simplex* (Rudolphi, 1809, det. Krabbe, 1878): Length distribution and viability of L3 of known minimum age from herring *Clupea*
*harengus* L. J. Helminthol..

[36] Monstad T., Monstad T. (1990). Some aspects of mortality, condition factors and liver state with Anisakis-infection in blue whiting in the North-East Atlantic. Proceedings of the Fourth Soviet-Norwegian Symposium, Bergen, Norway, 12–16 June 1990.

[37] Serrat A., Lloret J., Frigola-Tepe X., Muñoz M. (2019). Trade-offs between life-history traits in a coldwater fish in the Mediterranean Sea: The case of blue whiting *Micromesistius poutassou*. J. Fish Biol..

[38] Richards J. (1977). Preliminary results of the 1977 blue whiting surveys of the west of Scotland. Int. Counc. Explor. Sea.

[39] Rohde K. (1984). Ecology of marine parasites. Helgoländer Meeresunters..

[40] Mouritsen K.N., Hedeholm R., Schack H.B., Møller L.N., Storr-Paulsen M., Dzido J., Rokicki J. (2010). Occurrence of anisakid nematodes in Atlantic cod (*Gadus*
*morhua*) and Greenland cod (*Gadus*
*ogac*), West Greenland. Acta Parasitol..

[41] De la Torre Molina R., Pérez Aparicio J., Hernández Bienes M., Jurado Pérez R., Martínez Ruso A., Morales Franco E. (2000). Anisakiasis en pescados frescos comercializados en el norte de Córdoba. Rev. Esp. Salud Pública.

[42] Abattouy N., Valero A., Benajiba M.H., Lozano J., Martín-Sánchez J. (2011). *Anisakis**simplex* s.l. parasitization in mackerel (*Scomber*
*japonicus*) caught in the North of Morocco—Prevalence and analysis of risk factors. Int. J. Food Microbiol..

[43] Biary A., Berrouch S., Dehhani O., Maarouf A., Sasal P., Mimouni B. (2021). Prevalence and identification of *Anisakis* nematodes in fish consumed in Marrakesh, Morocco. Mol. Biol. Rep..

[44] Abattouy N., Valero-López A., Lozano-Maldonado J., Benajiba M.H., Martín-Sánchez J. (2014). Epidemiology and molecular identification of *Anisakis*
*pegreffii* (Nematoda: Anisakidae) in the horse mackerel *Trachurus*
*trachurus* from northern Morocco. J. Helminthol..

[45] Cipriani P., Acerra V., Bellisario B., Sbaraglia G.L., Cheleschi R., Nascetti G., Mattiucci S. (2016). Larval migration of the zoonotic parasite *Anisakis*
*pegreffii* (Nematoda: Anisakidae) in European anchovy, *Engraulis*
*encrasicolus*: Implications to seafood safety. Food Control.

[46] Farjallah S., Busi M., Mahjoub M.O., Slimane B.B., Paggi L., Said K., D’Amelio S. (2008). Molecular characterization of larval anisakid nematodes from marine fishes off the Moroccan and Mauritanian coasts. Parasitol. Int..

[47] Mattiucci S., Nascetti G., Cianchi R., Paggi L., Arduino P., Margolis L., Brattey J., Webb S., D’Amelio S., Orecchia P. (1997). Genetic and ecological data on the *Anisakis*
*simplex* complex, with evidence for a new species (Nematoda, Ascaridoidea, Anisakidae). J. Parasitol..

[48] Abollo E., Gestal C., Pascual S. (2001). *Anisakis* infestation in marine fish and cephalopods from Galician waters: An updated perspective. Parasitol. Res..

[49] Mattiucci S., Cipriani P., Levsen A., Paoletti M., Nascetti G. (2018). Molecular epidemiology of *Anisakis* and anisakiasis: An ecological and evolutionary road map. Adv. Parasitol..

[50] Hartwich G., Anderson R., Chabaud A., Willmott S. (1974). Keys to genera of the Ascaridoidea. CIH Keys to the Nematode Parasites of Vertebrates. No. 2.

[51] Yoshinaga T., Ogawa K., Wakabayashi H. (1987). Experimental life cycle of *Hysterothylacium* *aduncum* (Nematoda: Anisakidae) in fresh water. Fish Pathol..

[52] Petter A.J., Maillard C. (1988). Larves d’ascarides parasites de poissons en Méditerranée occidentale. Bull. Muséum Natl. D’histoire Nat..

[53] Zhu X.-Q., Gasser R.B., Podolska M., Chilton N.B. (1998). Characterisation of anisakid nematodes with zoonotic potential by nuclear ribosomal DNA sequences. Int. J. Parasitol..

[54] D’Amelio S., Mathiopoulos K.D., Santos C.P., Pugachev O.N., Webb S.C., Picanço M., Paggi L. (2000). Genetic markers in ribosomal DNA for the identification of members of the genus *Anisakis* (Nematoda: Ascaridoidea) defined by polymerase chain reaction-based restriction fragment length polymorphism. Int. J. Parasitol..

[55] Pontes T., D’Amelio S., Costa G., Paggi L. (2005). Molecular characterization of larval anisakid nematodes from marine fishes of Madeira by a PCR-based approach, with evidence for a new species. J. Parasitol..

[56] Bush A.O., Lafferty K.D., Lotz J.M., Shostak A.W. (1997). Parasitology meets ecology on its own terms: Margolis et al. revisited. J. Parasitol..

[57] Reiczigel J., Rózsa L. Quantitative Parasitology 3.0. Software 2005. https://www.zoologia.hu/qp/.

[58] Rózsa L., Reiczigel J., Majoros G. (2000). Quantifying parasites in samples of hosts. J. Parasitol..

[59] Reiczigel J., Marozzi M., Fábián I., Rózsa L. (2019). Biostatistics for parasitologists—A primer to Quantitative Parasitology. Trends Parasitol..

[60] Luna S.M. Dicologlossa cuneata (Moreau 1881) Wedge Sole. http://fishbase.org/summary/Dicologlossa-cuneata.html.

